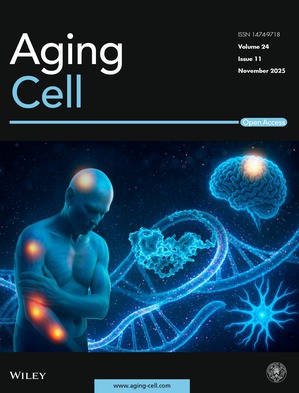# Featured Cover

**DOI:** 10.1111/acel.70291

**Published:** 2025-11-12

**Authors:** Rai‐Hua Lai, Ren‐Hua Chung, Wan‐Yu Pai, Yi‐Chung Chen, Ka‐Hei Lam, Cheng‐Nong Lai, Jyh‐Lyh Juang

## Abstract

Cover legend: The cover image is based on the article *Sex‐ and APOE Genotype–Dependent Pain Susceptibility and Alzheimer's Risk Mediated by the Lipid Metabolism Enzyme LPCAT2* by Rai‐Hua Lai et al., https://doi.org/10.1111/acel.70234.